# Thyroid Function Paremeters in Drug Naive, First Episode Mania: Are Thyroid Dysfunctions Present at The Onset of the Illness ? A Case Control Study

**DOI:** 10.1192/j.eurpsy.2025.646

**Published:** 2025-08-26

**Authors:** F. Çınar, Ö. Bayırlı, R. Tekdemir

**Affiliations:** 1 Psychiatry, Selcuk University, Konya, Türkiye

## Abstract

**Introduction:**

Bipolar disorder features recurrent episodes of mania, hypomania, and depression, with first-episode mania serving as an early indicator. The biological and psychological factors involved are not fully understood. Thyroid hormones play a vital role in brain metabolism, and their dysregulation has been linked to mood disorders, indicating potential abnormalities during manic episodes.

**Objectives:**

This study aims to evaluate the presence of thyroid dysfunction in drug-naive patients experiencing their first episode of mania compared to a healty control group.

**Methods:**

This study included forty-eight drug-naive patients diagnosed with first-episode mania, who were hospitalized for treatment, and a control group of forty-eight healthy individuals. The healthy control group was matched for age and sex and had no history of psychiatric illness or treatment. There were no physical illnesses in either group. Symptom severity was assessed using the Brief Psychiatric Rating Scale (BPRS) and the Young Mania Rating Scale (YMRS). Serum T3, T4, TSH levels were measured in both groups. The study protocol was approved by the Local Ethics Committee of Selcuk Üniversity.

**Results:**

There were no differences in sex and age distribution between the groups. In the patient group, 54.2% (n=26) were female, with a mean age of 24.98 (±7.38). Additionally, 16.7% (n=8) had a history of depression, and 49.7% (n=23) exhibited psychotic features. Analysis of serum TSH, T3, T4, and the T3/T4 ratio showed no significant differences between groups (TSH: t=-0.466, p=0.642; T3: t=1.258, p=0.212; T4: t=-0.874, p=0.382; T3/T4: t=-1.291, p=0.200). T3 levels were higher in males overall and in the control group (t=-3.000, p=0.004; t=-3.753, p<0.001), but not in the patient group (p>0.05). Among patients, T4 levels were significantly higher in those with psychotic features (t=-2.410, p=0.020). Correlation analysis showed no significant relationships between thyroid function tests and clinical variables.

**Conclusions:**

This study found no significant differences in thyroid function parameters between drug-naive patients with first-episode mania and healthy controls, suggesting that thyroid dysfunction may not be present at the onset of mania. While T4 levels were higher in patients with psychotic features, overall results indicate that thyroid abnormalities do not play a critical role in the immediate presentation of this disorder. These findings highlight the need for further research to explore the long-term relationship between thyroid function and bipolar disorder.

**Disclosure of Interest:**

None Declared

